# The Present and Future of the Poor-Law Infirmary

**Published:** 1913-04-05

**Authors:** C. Thackray Parsons

**Affiliations:** Medical Superintendent Fulham Infirmary.


### THE PRESENT AND FUTURE OF THE POOR-LAW INFIRMARY.

By C. THACKRAY PARSONS, M.D. Lond., Medical Superintendent Fulham Infirmary.

The provision of efficient medical and surgical treatment for the sick is a matter the importance and difficulty of which are becoming more and more generally appreciated. With increasing knowledge, treatment, both medical and surgical, has become more elaborate, necessitating the use of expensive apparatus, specialised buildings, and highly trained nurses. So much is this the case that for a large proportion of the population the major operations of surgery and the treatment of many acute and chronic diseases can no longer be efficiently carried out in the home. In England the consequent demand for institutional treatment has been met by the voluntary hospitals and the Poor-Law infirmaries. In theory whilst the former institutions are supposed to admit all applicants whose treatment is impossible under home conditions, the latter are supposed to confine themselves to the destitute sick. In practice, however, owing to various causes, of which the most important has been the inability of the voluntary hospitals to meet all the demands upon them, this division of cases has already completely broken down. In all Poor-Law infirmaries there are a large number of patients who are not destitute in the ordinary sense of the word, while the general voluntary hospital will not admit at all, or will admit only for a limited period, chronic cases even though they may need highly skilled nursing and specialised treatment quite unobtainable in the patients' homes. With the operation of the National Insurance Act a fresh factor of the greatest importance has been introduced. Even in these early days it has become obvious that institutional treatment will be required for a very large number of the insured—treatment for which the Act has made no provision whatever except in the case of patients in the early stages of tuberculosis. Sir Henry Burdett, in his illuminating address at the annual meeting of the Home Hospitals Association, estimated that in London alone probably 10,000 additional beds will be needed before long to meet this demand—a number approximately equal to that of the present accommodation of all the London voluntary hospitals. The solution he offers is a striking and bold one. He suggests that the accommodation required could be provided by the existing Poor-Law infirmaries if they were removed from the control of the Boards of Guardians and placed under the administration of a central body such as the Metropolitan Asylums Board. It becomes, therefore, of importance to consider how far the London Poor-Law infirmaries are carrying out efficiently the work already entrusted to them and what alterations are required to increase their efficiency and to enable them to deal with these new demands.

Probably very few persons outside those actively engaged in the work have any idea of the amount, complexity, and general excellence of the provision made for the institutional relief of sickness by the Poor-Law authorities in London. There are thirty-one Boards of Guardians in the county of London. These have under their control twenty-nine infirmaries which are completely separated administratively from the workhouses. The certified accommodation of these twenty-nine infirmaries is 16,615 beds, and, according to a return obtained by the Camberwell Board of Guardians, 15,503 of these beds were actually occupied on March 13, 1912. The medical and nursing staff of these twenty-nine infirmaries consisted of twenty-nine medical superintendents, sixty-seven assistant medical officers, twenty-nine matrons, ninety-three assistant matrons and night superintendents, 308 ward sisters, 1,642 staff nurses and probationers, and forty-one male nurses. In addition to these general infirmaries there are the specialised institutions of the Metropolitan Asylums Board, consisting of twelve fever hospitals with 6,528 beds, three smallpox hospitals with 2,040 beds, two hospitals for sick and convalescent children with 1,790 beds, three seaside homes partly for tubercular and partly for non-tubercular children with 384 beds, a ringworm school with 280 beds, two schools for ophthalmic children with 720 beds, and asylums and homes with 7,778 beds for imbeciles. With the exception of the fever and small-pox hospitals and the imbecile asylums practically all the cases admitted to these institutions are sent to them from the London Poor-Law infirmaries. In addition to all this, each of the London workhouses possesses separate wards used for the accommodation of the infirm and sick, the total number of beds thus used being approximately 9,000. The nature of the sick and infirm cases retained in the workhouses varies very much in different parishes. Speaking generally, only chronic and more especially senile cases are so retained. In some parishes the rule is that all patients who require to be kept in bed during the whole or part of the day are transferred to the infirmary, in others the workhouse accommodation is made use of for the treatment of bed-ridden, senile, and paralysed patients in whom there is little hope of improvement, venereal cases, and cases of ulcered legs. In a few parishes even acute cases are kept in the workhouse sick wards and the lying-in wards are found in the workhouse precincts.

It will be seen, therefore, that the provision made by the Poor Law in London for the institutional care of the sick poor consists of sick wards in workhouses, separate Poor-Law infirmaries, and the institutions of the Metropolitan Asylums Board. The system is seen at its worst in the sick wards of the workhouse. They are usually badly adapted for the purpose for which they are used, they are understaffed, and they are under the administrative control of the workhouse master, whose training and habit of mind are such as to render him in most cases totally unsuitable for the post. No system of reform will be complete which does not take into consideration these workhouse sick wards. On the other hand, the specialised hospitals of the Metropolitan Asylums Board are admirable institutions. They are well built, well planned, and in most cases well adapted for the purpose for which they are used. They are, as a rule, adequately staffed, and the subdivision of the cases amongst the different institutions enables the medical staff of each to specialise in the diseases they are called upon to treat. Even here, however, in the case of the general children's hospitals, the fever hospitals, and the imbecile asylums, special cases must often arise in which it would be of advantage if a specialist staff were available for consultation and for carrying out specialised treatment and methods of examination.

The position of the separate Poor-Law infirmary is a more difficult one to generalise upon, because of the vast differences which exist between individual infirmaries. They vary greatly in the type of building, in the adequacy of the staff, in the nature and age distribution of the cases admitted, in equipment, and in the character of the medical work performed within them. The different Boards of Guardians also vary in their attitude towards the infirmary they control, with results which depend to a certain extent upon the pliability or fighting powers of the medical superintendent. In some cases the Guardians are honestly anxious to do all in their power to provide the best treatment for the sick who come to them; in others they are only anxious to keep the expenditure as low as possible, and are indifferent as to the nature of the treatment provided so long as a public scandal is avoided. One has heard Guardians condemn an infirmary on the grounds that it was too efficient, and that its reputation attracted patients to seek admission to it. A medical staff under a controlling board actuated by considerations of this kind must always be hampered and prevented from doing its best work. The type of building varies very greatly. Those built of recent years—*e.g.*, the Bethnal Green, Islington, Hammersmith, and St. James's Infirmaries—are fine buildings, planned in accordance with the most modern views of hospital construction, and without excessive and unnecessarily expensive elaboration. Many of the older ones—*e.g.*, Camberwell, Hackney, and Marylebone—have been partly rebuilt, and now satisfy every requirement. Others possess glaring defects, which, however, only require the expenditure of a little money to put right; whilst a few are totally unsuitable for hospitals, and could be made suitable only by drastic reconstruction. As to the staffing, one finds this varies from one medical man to 220 patients to one medical man to 100 patients, as a rule the proportion being one medical man to about 125 patients. These numbers are calculated upon the number of patients in the infirmary; but it must be remembered that in most cases the medical staff of the infirmary also performs the medical work in the adjacent workhouse. As to the nursing staff, the numbers vary from one nurse (counting both day and night staff) to six patients to one nurse to fifteen patients, in most cases the proportion being about one nurse to eight patients. It will be obvious at once that as compared with the voluntary hospitals the staffing of the Poor-Law infirmaries—even the best of them—is very low. There is no doubt, indeed, that all the Poor-Law infirmaries are understaffed, and that some of them are scandalously understaffed. There can be no doubt, too, that this understaffing leads to inefficiency and to many cases being detained an unnecessarily long time in the infirmary.

The variation in the nature and age distribution of the cases admitted to the different infirmaries is also great. With regard to the nature of the cases admitted, it is difficult to speak with certainty, because detailed statistics are in most cases wanting, and one has to argue from such facts as the number of operations performed and the average length of stay of the patient in the infirmary—facts which may obviously be affected by other things than the character of the admissions. With regard to the age distribution, however, statistics I obtained from a few infirmaries some years ago showed a variation from 18 per cent. to 36 per cent. of patients over sixty-five years of age. With regard to operations under general anæthesia, in 1910 the number varied from twelve to 490, and as regards average length of stay of each patient this varies in different infirmaries from thirty-two days to 112 days.

There are also striking variations in the equipment of the different infirmaries, although the differences are not so great to-day as they were a few years back. Still, even to-day there are some infirmaries which do not possess an operating room and many in which the room provided is unsuited for the purpose; there are several without an *x*-ray apparatus; there are many without any apparatus for bacteriological or pathological examinations, and there are still more without any appliances for electrical treatment.

From this very brief review it will be seen how very great are the differences between individual infirmaries and how much below the best some of them are in construction, in equipment, and in efficiency. There is, however, one great defect which applies to all the London Poor-Law infirmaries as compared with the voluntary hospitals, and that consists in the absence of any specialist staff. In most of the infirmaries the medical superintendent specialises in some branch of his work, usually in operative surgery, and occasionally one of his junior medical officers may be specialising in some other branch, but it is obvious that this in no way meets the necessities of the case. To these infirmaries every variety of disease finds its way, and to be efficiently and properly equipped the same specialist staff should be available as at a voluntary hospital. It is obvious both for the sake of the patients and in the interests of real economy, the medical superintendent should be able to call in for consultation experts in any branch of medical or surgical work in which he knows he is not expert, just as he would do if he were on the staff of a voluntary hospital.

